# Optical metasurfaces towards multifunctionality and tunability

**DOI:** 10.1515/nanoph-2021-0684

**Published:** 2022-01-04

**Authors:** Kang Du, Hamdi Barkaoui, Xudong Zhang, Limin Jin, Qinghai Song, Shumin Xiao

**Affiliations:** Ministry of Industry and Information Technology Key Lab of Micro-Nano Optoelectronic Information System, Harbin Institute of Technology, Shenzhen 518055, P. R. China; Collaborative Innovation Center of Extreme Optics, Shanxi University, Taiyuan 030006, Shanxi, P. R. China

**Keywords:** multifunctional, optical metasurface, reconfigurable, tunable

## Abstract

Optical metasurfaces is a rapidly developing research field driven by its exceptional applications for creating easy-to-integrate ultrathin planar optical devices. The tight confinement of the local electromagnetic fields in resonant photonic nanostructures can boost many optical effects and offer novel opportunities for the nanoscale control of light–matter interactions. However, once the structure-only metasurfaces are fabricated, their functions will be fixed, which limits it to make breakthroughs in practical applications. Recently, persistent efforts have led to functional multiplexing. Besides, dynamic light manipulation based on metasurfaces has been demonstrated, providing a footing ground for arbitrary light control in full space-time dimensions. Here, we review the latest research progress in multifunctional and tunable metasurfaces. Firstly, we introduce the evolution of metasurfaces and then present the concepts, the basic principles, and the design methods of multifunctional metasurface. Then with more details, we discuss how to realize metasurfaces with both multifunctionality and tunability. Finally, we also foresee various future research directions and applications of metasurfaces including innovative design methods, new material platforms, and tunable metasurfaces based metadevices.

## 1 Introduction

The ability to control the wavefront of light is fundamental to focalization and redistribution of light, enabling many applications from imaging to spectroscopy. In recent decades, with the improvement of nanofabrication techniques, artificial nanostructures including photonic crystals and metamaterials have emerged. The early studies of metamaterial aimed to achieve abnormal effective medium coefficients and explore the corresponding anomalous physical phenomena by designing the meta-atoms and their spatial arrangement [1]. However, due to the high resonance loss and their 3D nature, the fabrication and the practical applications of metamaterials are limited. Different from the use of gradual phase accumulation to shape the light wavefront in metamaterials, metasurfaces can achieve two-dimensional (2D) control of light by introducing abrupt phase shifts along the optical path [2]. Notably, those metasurfaces can be fabricated by standard lithography and nanoimprinting techniques. Progressive research on metasurfaces has enormously expanded from plasmonic metasurfaces composed of metallic meta-atoms [3] to all-dielectric metasurfaces based on Mie resonance of high refractive index nanostructures [4]. Nowadays, metasurfaces are of great interest as a promising platform for holography [5], [6], [7], beam steering [8], structural color [9], [10], [11], [12], and other applications [13], [14], [15], [16].

Despite of many advances, limited working bandwidths and fixed wave-manipulation functionalities emerge as the main drawbacks in metasurfaces. In the case, many efforts have been devoted to achieving multifunctional and tunable metasurfaces that can dynamically control light upon external tuning. For example, one of the simplest multifunctionality strategies is to merge multiple monofunctional metasurfaces together by referring to the shared-aperture phase antenna [17]. This kind of multifunctional metasurface encompasses the benefits of multi-wavelength and angle-multiplexed holograms [18], [19], [20]. Another one is implemented by the integration of many similar or distinct functions into a single metasurface structure, which is similar to multiplexing in the field of telecommunication [21]. Moreover, several judiciously designed non-interleaved metasurfaces have been proposed to further improve the operation efficiency and the crosstalk [22]. In addition, the metasurface configurations based on supercell and multilayer share excellent performance in specific application fields, such as wide-angle optics [23] and achromatic metalens [24]. Note that seeking more degrees of design freedom is the fundamental way to develop multifunctional metasurfaces [21, 25, 26].

As for the tunability, it can be commonly addressed by combining static metasurfaces with active materials [27], [28], [29], [30], [31], [32], therefore leading to that the optical response of such metasurfaces can be controlled by altering the effective permittivity of the active material via external stimuli. Obviously, studies on the characteristics of material platforms will directly drive the development of tunable metasurfaces [33], [34], [35]. It is also worth mentioning that, based on the goal of reconfigurability, the concept of digital coding metasurface has been widely developed in GHz and THz waves, which is extremely important for realizing the full-space and space-time manipulation of electromagnetic waves, and also contributes to the realization of the intelligent metasurfaces [36, 37]. Nevertheless, such concepts are challenging to be implemented in the visible region due to the lack of appropriate strategies and active materials.

The development of optical metasurfaces with multifunctionality and tunability are not independent, both of which aim to promote industrialization for metasurfaces. Nevertheless, the challenges, together with the pressing demands from application side, drive this field move forward quickly and have made it a frontier in metasurface research. Several review papers have come out with different priorities, such as material platforms [38], [39], [40], [41], [42], [43], [44], [45], [46], [47], [48], specific functions [9, 10, 49], [50], [51], [52], [53], [54], [55], [56], [57], [58], and design methods [25, 26, 59], [60], [61]. There are also long articles [62], [63], [64] that comprehensively introduce the development of the field. In this review, we will outline some common ways to construct multifunctional metasurfaces in Section 2. Afterwards, we will briefly describe in Section 3 the tunability and reconfigurability induced by different external stimuli. In Section 4, we highlight a few studies on metasurface with both multifunctionality and tunability in the visible range. Finally, we share some thoughts on the future development of this field.

## 2 Multifunctionality from the pattern design

Polarization [65], [66], [67], [68], [69], wavelength [20, 24, 69], [70], [71], and angle [72], [73], [74], [75] of incidence are part of a non-exhaustive list of parameters of a given light source that are usually taken advantage of as multiplexing channels for the multifunctional metasurfaces [21], which realize holography, structural color, and beam steering at the same time. The schemes for multifunctional metasurface design can be roughly divided into two categories. One is to simply merge the individually designed metasurfaces with different functions to form a multifunctional metasurface in segmented or interleaved configurations [17, 76]. The other is to use linear property of the Fourier transform to add the complex transmission profiles and meet the requirement for multiple functions in one time [17, 23, 77]. Recently, by introducing the concept of supercell and considering the non-local effects, the second scheme has been extended and realizes series of wide-angle optical elements [23]. Furthermore, designing multilayer metasurfaces does not only achieve specific functions more efficiently, such as combining functional metadevices with vastly different operating wavelengths [78], but also further broaden the application range of metasurfaces under the guidance of ingenious ideas [79].

### 2.1 Segmented and interleaved metasurfaces

Capitalizing the shared-aperture phased antenna array technology widely adopted in radar applications, photonic metadevices can be made multifunctional [17, 80]. In the early stage, the majority of multifunctional metasurfaces were developed by this kind of method, that is, simply segmenting or interleaving two or more individually designed metasurfaces with similar or distinct functions [21, 76]. Although the distribution patterns of the metasurfaces with different functions appear on the same metadevice, the realization of different functions still depends on different channels due to independent design. Below, we will show two examples illustrating how to switch the functions of metasurfaces through the wavelength or polarization of incident light.

**Figure 1:**
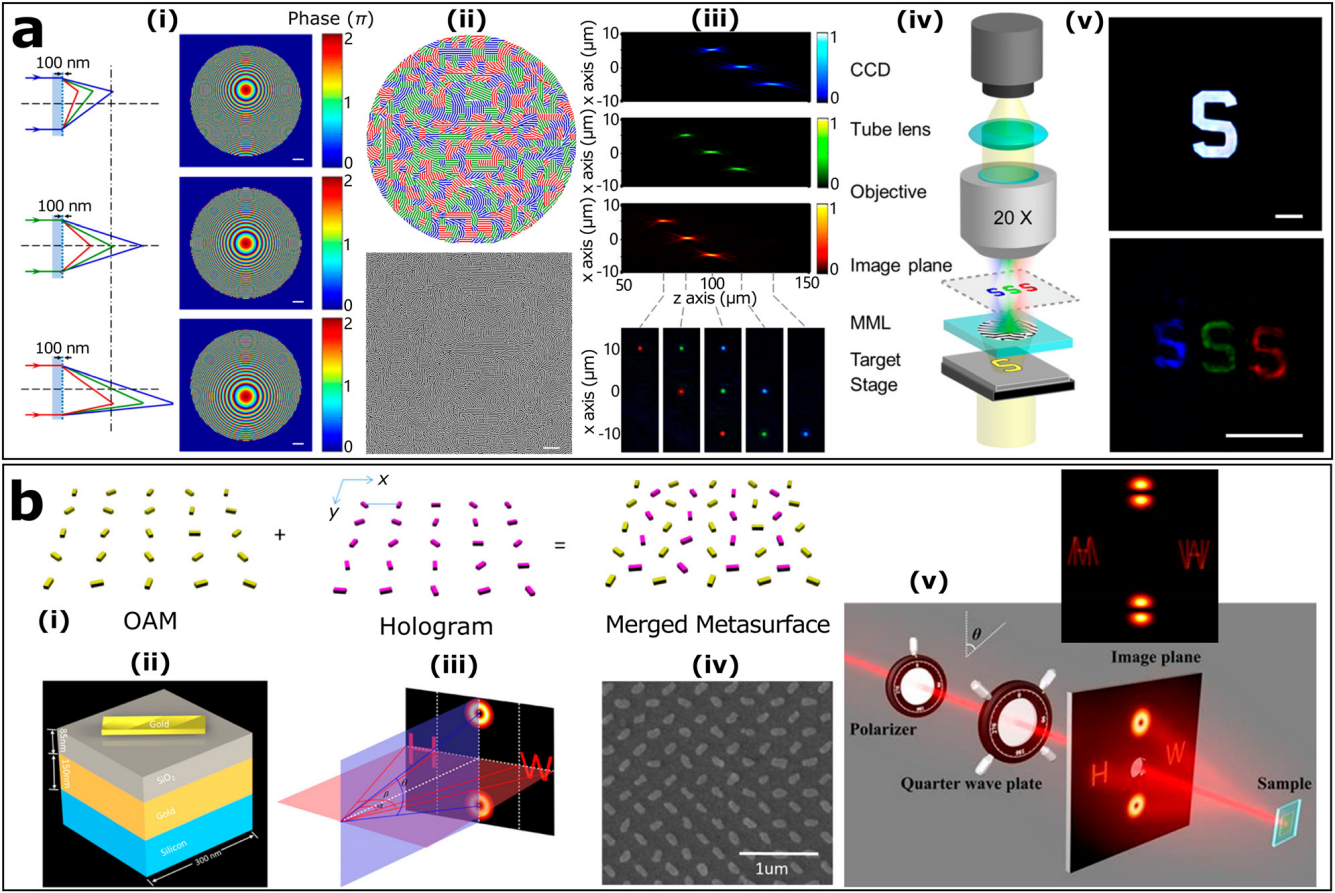
The segmented and interleaved metasurfaces. (a): (i) Schematic of light rays focused and steered by individual metalens and the corresponding phase profiles. (ii) Nanopattern design of a multifunctional metasurface that incorporates the functions of three metalenses, and the scanning electron microscope (SEM) image of the fabricated multifunctional metalens. Scale bar: 2 μm. (iii) Measured intensity profile behind the multifunctional metalens in the *x*–*z* plane and *x*–*y* plane at three different wavelengths. (iv) Schematic of the optical microscope setup using the fabricated multifunctional metalens. (v) Optical microscopy image of the target, and the image generated by the color separating metalens. Reproduced with permission from ref. [20]. Copyright 2016, American Chemical Society. (b): (i) Design schematic of the multifunctional metasurface. (ii) Schematic of the unit cell. (iii) Geometric parameters of the projected holograms and OAM beams. (iv) SEM image of the fabricated metasurface. (v) Schematic of simultaneous control of holograms and superposition of OAM. Reproduced with permission from ref. [81]. Copyright 2017, American Chemical Society.

The axial and lateral multifocus metalens can be created by interleaving three distinct metalenses that have a shared focal length and laterally separated focal spots at different operating wavelengths [20]. The phase profiles of the three metalens are designed to focus and steer light with different wavelength and are implemented as Si-based gradient metasurfaces. These metalenses, whose phase profiles are non-centrosymmetric, can be randomly divided into segments with areas 600 × 600 nm^2^ by a square lattice arrangement. The final designed nanopattern combining the optical functions of the three metalenses is shown in Figure 1(a). The imaging system based on this multifunctional metalens can perform focusing and color separation simultaneously and only shows a relatively weak intensity of focused light without sacrificing the spatial resolution [20, 76].

In addition, segmented and interleaved metasurfaces are widely exploited in polarization-controlled multifunctional metadevices [21, 62]. An ultrathin optical metadevice based on interleaved metasurface has been experimentally confirmed to concomitantly realize controllable holograms and superposition of orbital angular momentum (OAM) through different polarizations of incident light [81]. As shown in Figure 1(b), two metasurfaces designed to operate with opposite incident helicities carry the signal of two holographic images along the horizontal direction and two OAM beams along vertical direction. They are merged to form the multifunctional metasurface by moving one of them along the displacement vector of (*d*/2, *d*/2), where *d* is the lattice constant. Now, the different superpositions of OAM states and different holographic images can be reconstructed by controlling the polarization state of the light illuminated on the multifunctional metasurface. The combination of functionalities and channels will lead to a significantly increase of the density of function as well as the decrease of the size of the device [81].

These multifunctional metasurfaces constructed through a merging scheme are easy to design and can be directly applied to achieve the integration of many other functions [18, 19, 82], [83], [84], [85], [86], [87], [88], [89], [90]. Recent studies on multifunctional metasurfaces have further shown that the optical response of the improved interleaved metasurfaces can be simultaneously and independently manipulated by controlling the arbitrary optical parameters of incident light, including wavelength, polarization, and incident angle [87]. So many degrees of freedom for design can significantly expand the application fields of multifunctional metasurface. Nevertheless, the multifunctional metadevices based on segmented and interleaved metasurfaces will still be severely affected by functional crosstalk. Besides, the extremely low operational efficiency (approximately limited to 1/*N*, where *N* is the number of functions) will seriously restrict their practical application [20, 23]. Optimizing the efficiency of meta-atoms and the arrangement of interleaved cells are fairly the best methods to further improve the performance of such multifunctional metadevices.

### 2.2 Non-interleaved metasurface

**Figure 2:**
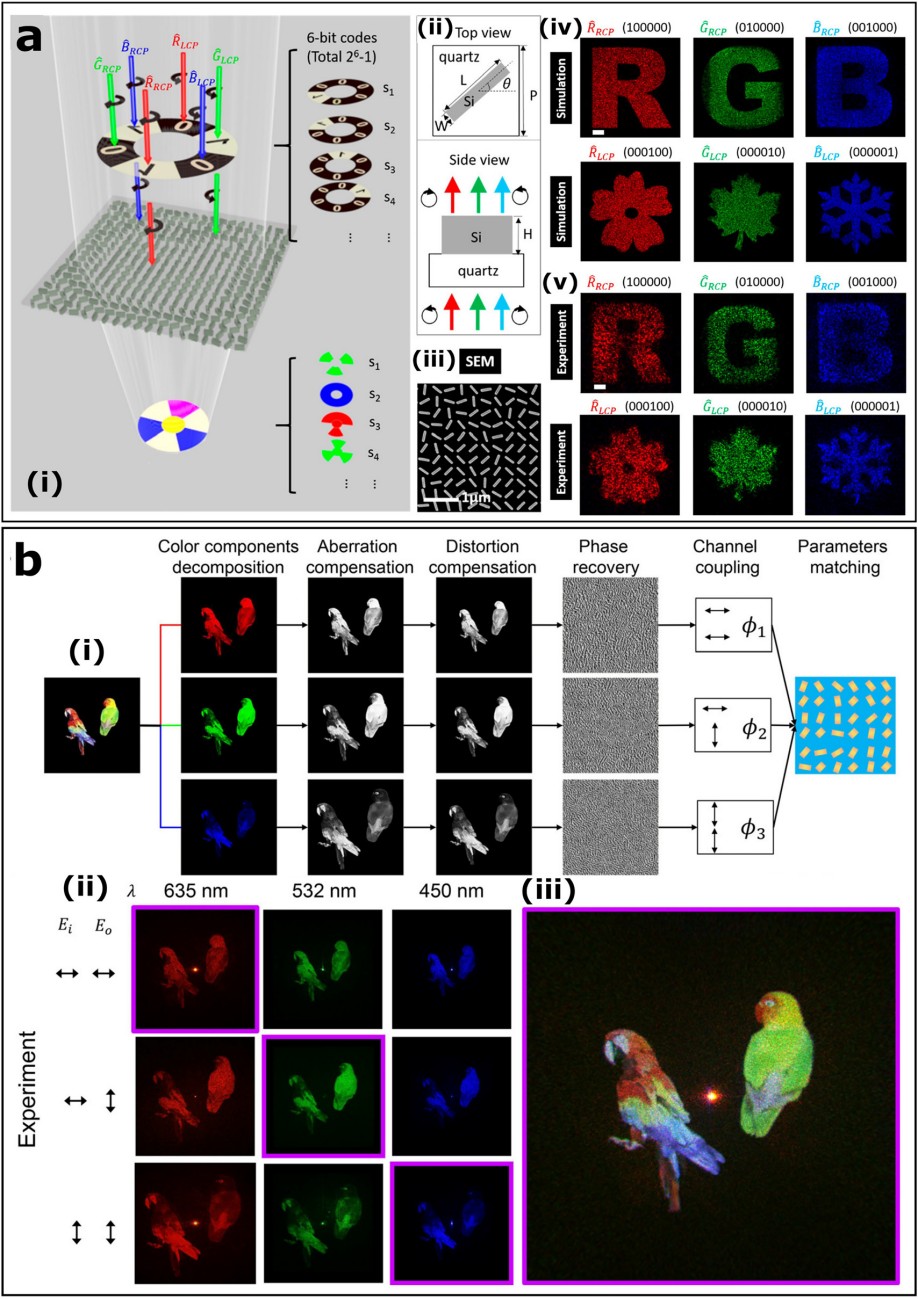
The non-interleaved metasurfaces. (a): (i) Schematic of the transmission-type 6-bit metasurface. The wavelengths with spins of input beams represent the fundamental bases. (ii) Geometry of the designed unit cell. (iii) SEM image of a partial region of the fabricated Si metasurface. Simulated (iv) and experimental (v) reconstruction of holograms from the designed multifunctional metasurface. Reproduced with permission from ref. [70]. Copyright 2018, American Chemical Society. (b): (i) Design process of vectorial color holographic metasurface. (ii) Experimental hologram images projected with all channels. (iii) The composed vectorial full-color hologram image. Reproduced with permission from ref. [71]. Copyright 2019, American Chemical Society.

In order to solve the functional crosstalk and inefficiency problems of segmented and interleaved metasurfaces, a variety of multifunctional metasurfaces based on non-interleaved nanopattern have been proposed [17, 22, 70, 71, 77, 91], [92], [93], [94], [95]. Finding more independent channels and one-time design scheme are common ideas for solving crosstalk and inefficiency challenges. For instance, the off-axis technique is applied to multiplex and demultiplex multiple OAMs on highly integrated metasurfaces [91]. Figure 2(a) shows another example of a multifunctional metasurface which is capable of 6-bit encoding color holograms by manipulating spin and wavelength of incident light at the same time [70].

According to the different manipulated physical quantities, the schemes for designing the multifunctional metasurfaces are also different [17, 77]. In general, the target far-field distribution of the multifunctional metasurface is a Fourier transform function of its complex transmission profile [21, 23, 76]. The relationship can be mathematically expressed as

$$E_{1}+E_{2}+\cdot \cdot \cdot =ℱ\left\{E_{inc}\left(x,y\right)\cdot T_{meta}\left(x,y\right)\right\}$$

where *E*_i_ and *E*_inc_ indicate the far-field distribution corresponding to the individual function and the incident field distribution, respectively, and *T*_meta_ is the complex transmission profile of the multifunctional metasurface. Therefore, the complex transmission profile of each function can be directly added up thanks to the linearity of the Fourier transform.

This approach is intuitive for solving the energy-tailorable questions [92] and can also be easily extended to polarization optics [71, 77, 93, 96] with the help of the dielectric anisotropic meta-atom which have the ability to control phase and polarization simultaneously [22]. Recently, it has been confirmed that since the broadband phase profiles are encoded into three orthogonal polarization bases, the non-interleaved TiO_2_ metasurface has almost zero crosstalk for reconstructing trichromatic holography [71]. The design process of this vectorial trichromatic meta-holography can be seen in Figure 2(b), where the aberration and distortion compensations are used to deal with the size mismatch of the three-component hologram caused by chromatic aberration and the distortion caused by the large field of view, respectively. The high-quality color holography demonstrated through the experiment will allow the multifunctional metasurfaces to take a big step towards the practical application [71]. Generally, the metasurface obtained by this method will have a better signal-to-noise ratio and higher function density.

### 2.3 Non-local supercells

The two multifunctional metasurface design schemes described in the above section used a pre-calculated library of meta-atoms which is independent. Indeed, the meta-atoms’ transmission phase simulation mainly embrace the assumption of Bloch or periodic boundary conditions, meaning that the mutual coupling effect between meta-atoms is completely ignored. Since non-local effects are not considered, the multifunctional metasurface designed according to the above two schemes will severely limit the realization of wide-angle function [23, 97, 98]. Specifically, a large phase gradient is required if the metasurface can deflect beam at large-angle. However, the adoption of the common local meta-atoms in such case will probably cause under-sampling, significant efficiency loss or other undesirable impacts.

**Figure 3:**
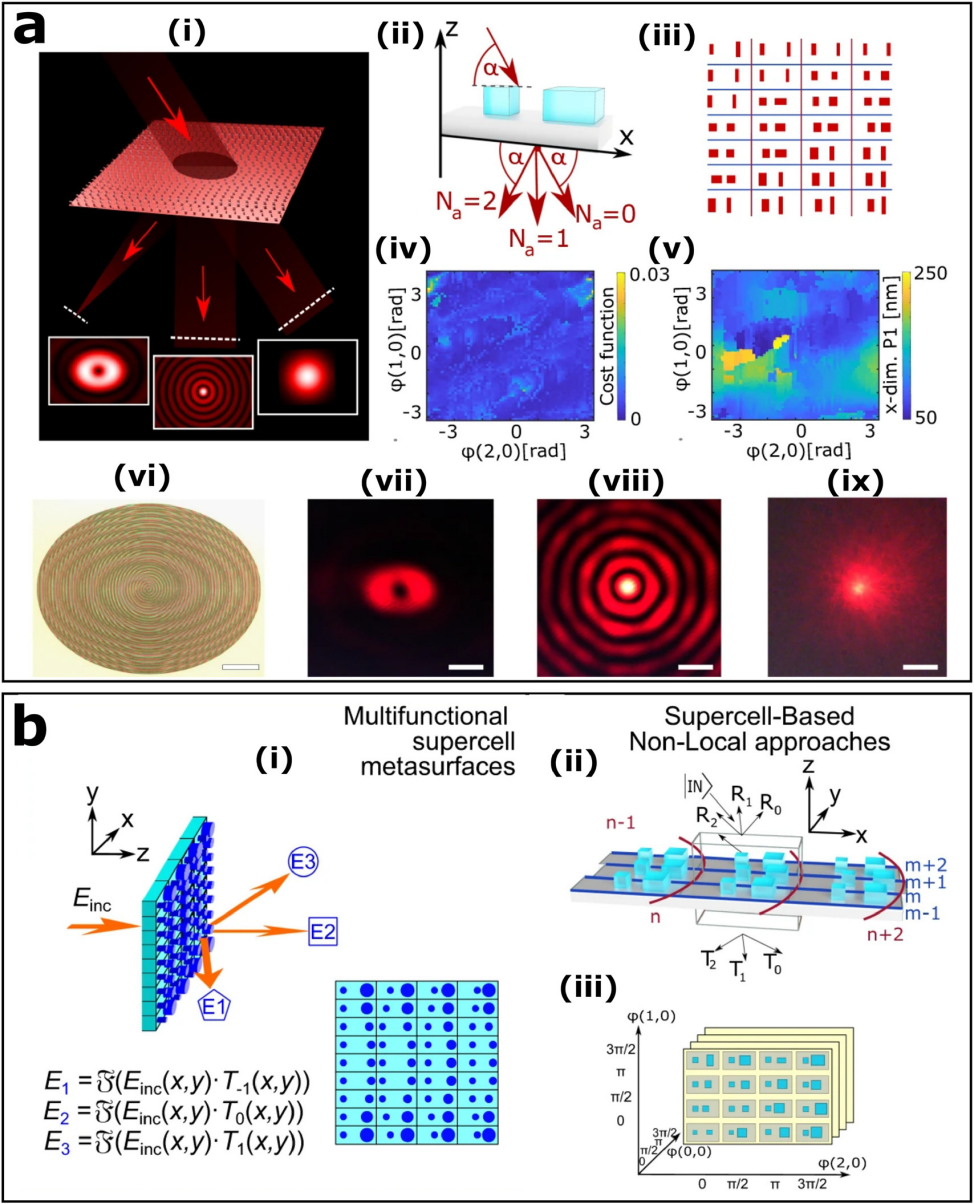
Multifunctional beam shaping based on supercell metasurface. (a): (i) Schematic of the multifunctional beam shaping metasurface. (ii) Schematic of the two-pillar supercell. (iii) The configuration of the metasurface. (iv, v) The optimization results of the supercell library showing different parameters for a 2*π* phase coverage on the orders (1, 0) and (2, 0). (vi) Optical image of the fabricated metasurface. Scale bar: 165 µm. Optical measurements of the focused helical beam (vii), the Bessel beam (viii), and the 0th order in the far field (ix). Scale bar: 8 µm. (b): (i) The generalized supercell metasurface concept. The far-field corresponding to each function is associated through the fourier transform to the phase profile loaded on each order. (ii) Nonlocal supercell holds multiple diffraction orders by considering the coupling between the elements. (iii) Schematic of multidimensional library of non-local supercells. Reproduced from ref. [23] under a Creative Commons Attribution 4.0 International License, Springer-Nature.

Inspired by the concept of meta-grating, the meta-atoms in a metasurface are replaced by the supercells in a number of recent studies in order to explore the efficient large-angle scattering [23, 99], [100], [101], [102], [103], [104]. The multiple diffraction orders induced by the supercells will bring more design degrees of freedom and overcome the local effect in the design of the metasurface to a certain extent. For instance, Capasso’s research group adopted an extended supercell method to design a highly efficient metasurface with multiple independent optical functionalities at an arbitrary large deflection angle [23]. Figure 3(a) shows the designed multifunctional beam shaping metasurface based on the supercells containing two pillars which are able to split the incident Gaussian beam into a Gaussian, a Bessel, and a focused helical beam at different deflection angles. The extended supercell is a platform that contains multiple adjacent meta-atoms and considers the non-local effects caused by the coupling between them. The redistribution of optical power within a non-local supercell will enable the local transmittance or reflectance to exceed unity. Furthermore, as shown in Figure 3(b), the multidimensional library of non-local supercells contains the corresponding phases and amplitudes on each diffraction order in addition to a list of geometries of supercells.

The non-local effects of metasurfaces can not only allow spatial modulation owing to multiple diffraction orders, but also render strong frequency selectivity because of high-Q resonances [97, 98, 105]. Combining these effects with the local metasurfaces that mainly manipulate the wavefront will surely provide a unique platform for the development of multifunctional nonlinear optical metadevices.

### 2.4 Multilayered metasurfaces

The optical metasurface significantly eases the requirements for nanofabrication technology. Moreover, it drastically reduces the degree of design freedom. Hence, it is more challenging for design approaches and optimization techniques to implement complex functionalities with a single layered metasurface. Indeed, this will lead to huge consumption of computing resources and time. To solve the above problems and further promote the application of metasurface, many efforts have been made on the topic of multilayered functional metasurfaces [59]. Especially in the case of wavelength-selective or broadband multifunctional metadevices, such as achromatic or zoomable metalenses [24, 49, 106], the configuration of multilayered metasurfaces has unique advantages [21].

**Figure 4:**
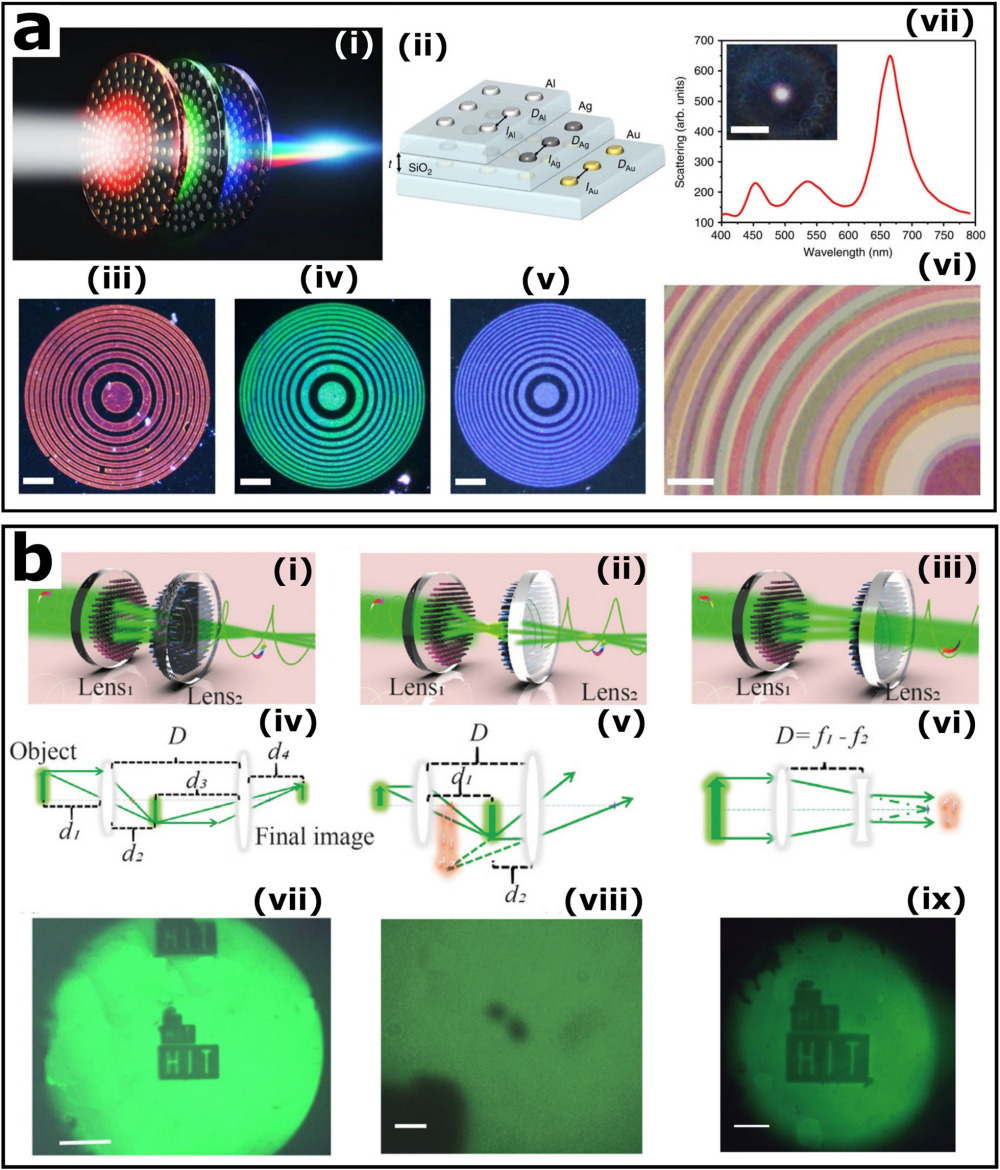
Multilayered metasurfaces. (a): (i) A rendered picture of the three-layer lens. (ii) Schematic of the layered structure. (iii–v) Dark-field images of the single-layer lens elements. Scale bar: 35 µm. (vi) Bright-field transmission image of the three-layer lens. Scale bar: 10 μm. (vii) White focal spot and corresponding spectrum. Scale bar: 20 µm. Reproduced from ref. [24] under a Creative Commons Attribution 4.0 International License, Springer-Nature. (b): The schematics of the metalens doublet for the functions of a camera lens (i), a compound microscope (ii), and the Galileo telescope (iii). The corresponding 2D schematics (iv–vi) and the final images (vii–ix) are illustrated in bottom panels of (i–iii). Scale bar: (vii) 30 µm, (viii) 3 µm, (ix) 100 µm. Reproduced with permission from ref. [79]. Copyright 2020, WILEY-VCH.

Avayu et al. demonstrate an achromatic multilayer lens in the visible range using a dense vertical stacking of three independent metasurfaces. Each layer was made of different metal designed to optimally interact with light at three different wavelengths in the visible range [24]. As shown in Figure 4(a), each metasurface acts as a Fresnel zone plate with a narrow band and focuses the corresponding light to the shared focal point. Recently, a spin angular momentum (SAM) controlled multifunctional metalens doublet as shown in Figure 4(b) has been reported [79]. First, they designed and fabricated two different polarization-controlled TiO_2_ metalenses. Starting from two convex lens functions, switching the function of one of the metalenses from convex to concave one by changing the handedness of incident light. The combination of two metalenses, by controlling the SAM of the incident light and the spacing between them, can play the functions of a camera lens, a compound microscope, or a Galileo telescope [79].

Furthermore, the extra degree of freedom always leads to new physical phenomena. If a non-zero rotation angle is introduced between the different layers of stack metasurfaces, the formed twisted metasurface will show an optical chiral response [59]. The research of multilayered metasurfaces and other related fields, such as the twist-stacked multilayer 2D nanomaterials [107] and Moiré Metasurfaces [108], are all worthy of attention. It may also be noted that if multilayered metamaterials are used for optically encrypted reprogrammable meta-holograms [7], their security level can be further improved.

## 3 Tunability from external stimuli

In addition to integrating multiple functionalities, achieving dynamic tunability is also a primary issue for metasurfaces. Therefore, the creation of nanostructures that can quickly respond to external stimuli is another research highlight in recent years, and this is closely connected to natural active materials. The optical response of metasurfaces, either by combining active material within static metasurface or by directly fabricating metasurface using active material, can be dynamically manipulated by external stimuli [25], [26], [27], [28], [29], [30], [31], [32], [33], [34], [35, 38], [39], [40], [41], [42], [43], [44], [45, 109], [110], [111], [112], [113]. In most studies, active materials mainly involve transparent conducting oxides (TCOs), phase change materials (PCMs), two-dimensional materials (2-DMs), liquid crystals (LCs), etc. [112, 113]. The external stimuli principally include electrical, optical, thermal, magnetic, and chemical stimuli [28, 112]. Most experiments that manipulate the optical response through external stimuli are based on tuning of the dielectric constant of active materials, whereas the systems tuned by mechanical deformation or reconfigurable design directly alter the interactions between units of metasurfaces. In addition, the ultrafast optical nonlinear process induced by strong light pumping provides more control freedom for tunable devices and offers the basis for realization of ultrafast switching, signal processing, frequency conversion, and pulse generation.

### 3.1 Stimuli to change the permittivity

#### 3.1.1 Electrical and optical stimuli to TCOs/PCMs/2-DMs

The most common external stimuli include electrical, optical, and thermal stimulations. Different stimuli to the active material often induce different responses. Therefore, the selection of the most effective stimuli according to the characteristics of the active material itself is the basic principle of designing tunable metasurfaces. For example, vanadium dioxide (VO_2_) can easily switch between insulator and metal by controlling the temperature, so thermal-controlled VO_2_-based metasurfaces are the most prevalent [31, 40]. However, the ordinary heating process can only make amorphous Ge-Sb-Te (GST) crystallization, while its reverse process must be achieved by other methods, such as optical or electrical pulses [114], [115], [116], [117]. For most TCOs, PCMs and 2-DMs, external electrical and optical stimuli ease the control of their optical responses by changing the free carriers inside the materials.

**Figure 5:**
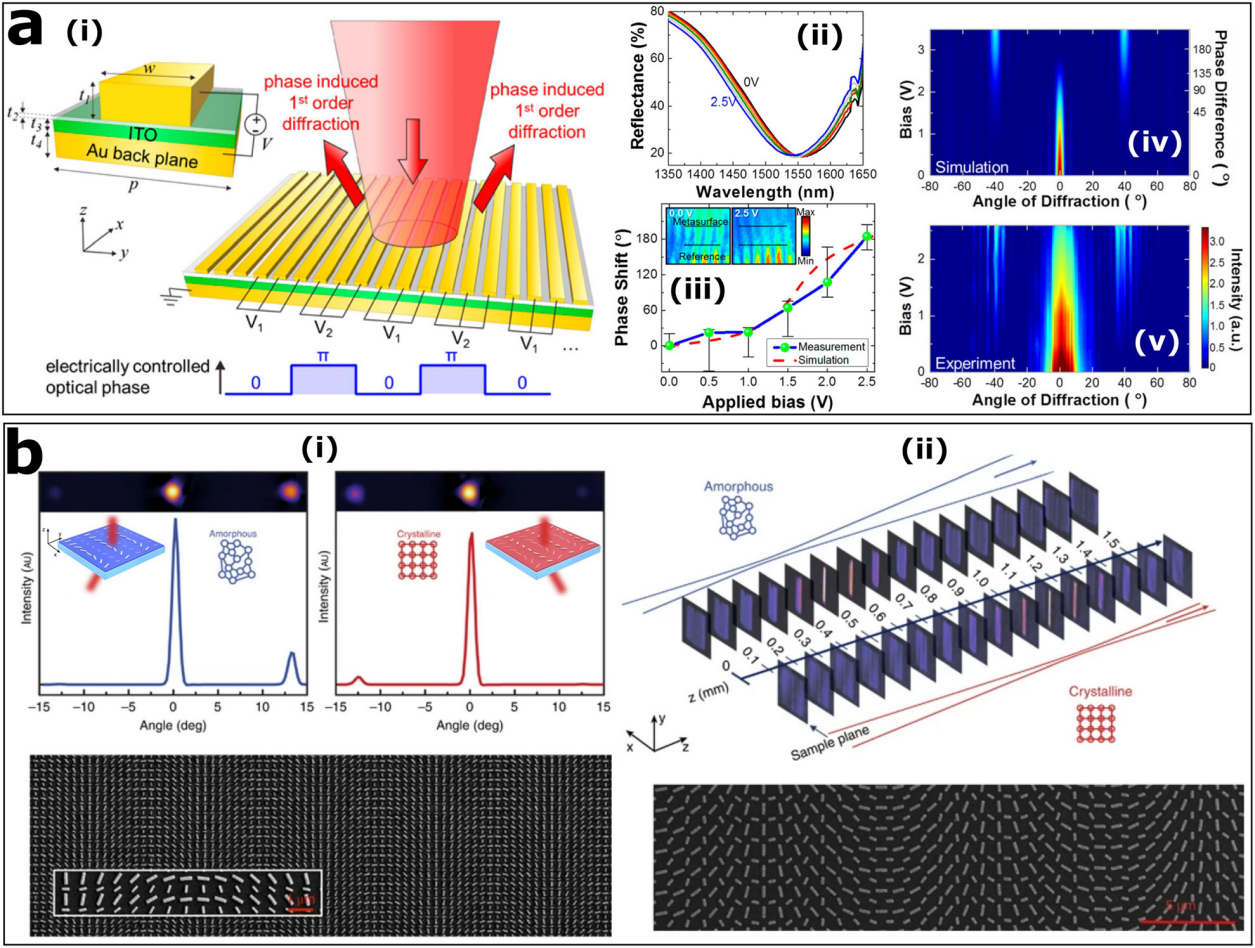
ITO-based and GST-based tunable metasurfaces. (a): (i) Schematic of the gate-tunable metasurface. (ii) Measured reflectance spectrum from the metasurface for different applied bias. (iii) Measured and simulated phase shift as a function of applied bias. (iv) Simulated and (v) experimental measured far-field intensity profiles of the diffracted beam versus applied bias. Reproduced with permission from ref. [124]. Copyright 2016, American Chemical Society. (b): (i) Tunable plasmonic metasurface for beam switching. Top: Infrared camera images and intensity plots of the beam transmitted by the metasurface in the amorphous (left) and crystalline (right) state. Bottom: SEM image of the metasurface. Scale bar: 1 µm. (ii) Tunable plasmonic metasurface for bifocal zoom. Top: Camera pictures of the cylindrical bifocal lens imaged at different distances *z*. Bottom: SEM image of the metasurface. Scale bar: 5 µm. Reproduced from ref. [116] under a Creative Commons Attribution-NonCommercial-NoDerivs 4.0 International License, Springer-Nature.

Indium tin oxide (ITO), as a typical TCO material, has received a lot of attention in recent years [118], [119], [120], [121], [122], [123], [124], [125], [126]. It actually exhibits some exotic phenomena at the epsilon-near-zero (ENZ) wavelengths and has considerable application potential in ultrafast switching. Therefore, the modulation speed of the ITO-based metadevice is of concern. For an ITO thin-film of 310 nm, the modulation time can be as short as 650 fs, which is proved by a degenerate pump-probe experiment at ENZ wavelengths [119]. An optical nonlinear metasurface based on the coupling of gold dipole antennas and ITO, the ENZ material, was later proposed to offer broadband and ultrafast nonlinear refractive index [122]. Its modulation time is about 1 ps, which is longer than that of ITO itself. Furthermore, as shown in Figure 5(a), a gate-tunable metasurface based on field-effect modulation of the complex refractive index of ITO layers enables dynamic electrical control of the phase (∼184°) and amplitude of the reflected wave [124]. The modulation speed of up to 10 MHz was experimentally demonstrated. Generally speaking, it is difficult for the modulation speed of metadevices to exceed that of active materials, and the most significant influence comes from the *Q*-value of nanostructures. Additionally, all-optical modulation is also faster than electro-optical modulation since there are no bandwidth and capacitance limitations in the former case [112].

The application of metasurfaces based on PCMs, especially GST-based metasurfaces, to non-volatile analog memory has attracted a lot of attention recently, mainly due to their ability to be combined with neuromorphic photonic processors [127, 128]. In addition, there are many other applications for GST-based metasurfaces as depicted in Figure 5(b). The two metasurfaces for beam switching and bifocal lensing employ a combination of resonant plasmonic antennas and a GST layer [116]. The tunability of the metasurfaces depends on the switching of GST layer between the crystalline and amorphous states. It is also well-known that the phase transformation of GST thin-film can be reversibly achieved on ultrafast time scales by picosecond laser pulses, whereas the cycle durability is limited [115]. Therefore, it is worth exploring the method to improve cycle life in practical applications [129].

Graphene, a typical 2-DM, is mainly applied on tunable THz metasurface. On one hand, applying an electrical stimulus on graphene metasurface can lead to a fast modulation speed [31, 40, 130]. On the other hand, it can also be used for optical-controlled metasurfaces. For instance, the plasmonic resonances of graphene can be tuned by ultraviolet (UV) illuminations [131]. As for LCs, its combination with metasurfaces opens a new direction for the study of tunable metasurfaces [47, 132], but the modulation speed of relevant metadevices is limited due to the slow response time of LC itself [133]. Nevertheless, the prototype of addressable LC-based metasurfaces [134] will bring more possibilities for various dynamic reconfiguration applications as discussed in Section 3.2.

#### 3.1.2 Surrounding medium

Tuning dielectric constant of active materials via external stimuli is always limited by the intrinsic characteristics of the studied media. For example, even at ENZ wavelength, the absolute refractive index change of ITO caused by optical pump is smaller than that of GST caused by a phase transition in the near-infrared region [119, 135]. Therefore, altering the surrounding medium of functional metadevices is a more direct and less restrictive method, which has noticeable advantages in the dynamic control of structural color based on all-dielectric metasurface.

**Figure 6:**
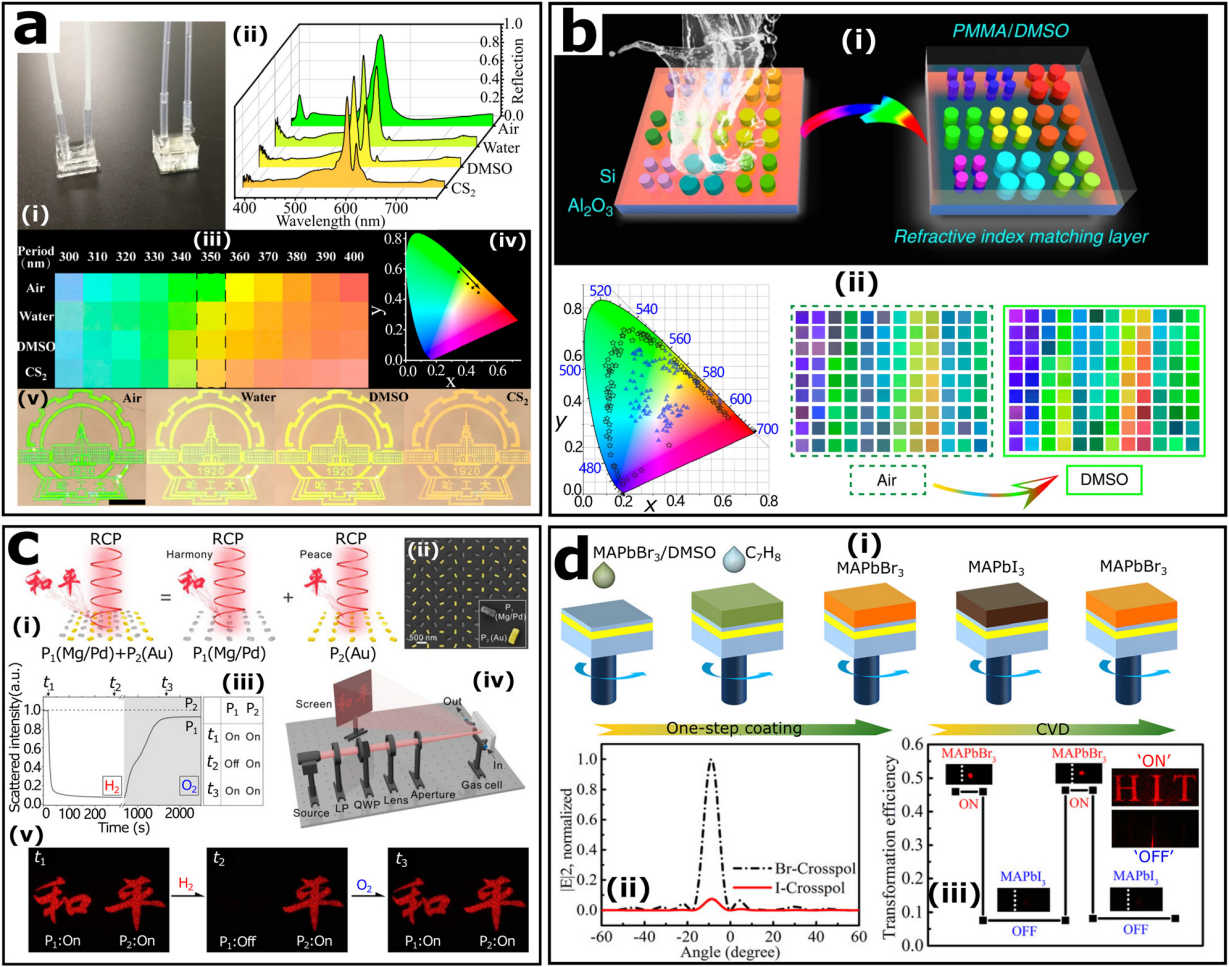
Tunable metasurfaces based on altering surrounding medium and material composition. (a): (i) The tunable metadevices integrated TiO_2_ metasurfaces with microfluidic chips. (ii) Experimental reflection spectra of the metasurface (period: 350 nm) surrounded by different solvents. (iii) Reflection colors of metasurfaces with different periods recorded under a bright-field microscope. (iv) Chromatic coordinates of the colors in (ii) marked in the CIE 1931 color space. (v) Color images of the school badge of Harbin Institute of Technology composed by the TiO_2_ metasurface. Reproduced with permission from ref. [138]. Copyright 2018, American Chemical Society. (b): (i) Schematic of improving the performance of structural color of the Si metasurfaces by covering a refractive index matching layer. (ii) Experimentally recorded color palettes of 108 Si metasurfaces in air (triangles) and in dimethyl sulfoxide (DMSO, stars). Reproduced from ref. [12] under a Creative Commons Attribution 4.0 International License, Springer-Nature. (c): (i) Two holographic patterns reconstructed from two independent phase profiles containing dynamic Mg/Pd (P_1_) and static Au (P_2_) nanorods. (ii) SEM image of the hybrid plasmonic metasurface. (iii) The scattering intensities of P_1_ and P_2_ varies with time during hydrogenation and dehydrogenation. (iv) Schematic setup for holographic reconstruction. (v) Representative snapshots of the holographic images. Reproduced from ref. [139] under a Creative Commons Attribution NonCommercial License 4.0, American Association for the Advancement of Science. (d): (i) Schematic pictures of synthesis process. (ii) The calculated far field angular distributions of anomalous reflection from MAPbBr_3_ metasurface and MAPbI_3_ metasurface. The experimentally recorded anomalous reflection (iii) and holographic image “HIT” (inset) in “ON” and “OFF” states. Reproduced with permission from ref. [140]. Copyright 2019, WILEY-VCH.

Compared with Mg [42] and Pb [136, 137], which are regularly exploited in dynamic structural color of plasmonic metasurfaces, all-dielectric materials, such as Si and TiO_2_, have more stable properties, so it is difficult to tune their optical properties directly by an external electrical or optical stimulus. However, considering the extreme sensitivity of Mie resonances of dielectric nanostructures to the surrounding refractive index, it is feasible to dynamically alter the structural color of the all-dielectric metasurface [9, 10] by directly varying the surrounding medium. Figure 6(a) presents the real-time tunable structural colors which were achieved by embedding the TiO_2_ metasurface into a polymeric microfluidic channel [138]. Altering the refractive index in a microfluidic channel allows a concise control of the reflection spectra of a TiO_2_ metasurface and its corresponding colors. Besides, the tunable colors are able to span the entire visible range by simply changing the lattice constant of the metasurface. Two years later, the same research group proposed that the structural color performance of the Si metasurface could be substantially enhanced by adding a refractive index matching layer [see Figure 6(b)]. This extra-layer is capable of effectively suppress the reflection from the substrate and narrow the bandwidth of reflection spectrum at the same time [12]. The experiments demonstrated that the gamut of the proposed metasurface has been increased to around 181.8% of sRGB and the distinct structural color can be preserved even in a small pixel with 2 × 2 array of nanostructures. At this point, structural color will display large-gamut, high-brightness, and high-resolution.

#### 3.1.3 Change of the material composition

Chemical or electrochemical reactions, such as ion implantation technology or hydrogenation reaction, can appropriately alter the composition of certain materials, resulting in an adjustment of their refractive index. This process widens the approaches for realizing the dynamic tunability of metasurfaces. Early experiments mainly focused on the transformation of metal materials, typically magnesium, to dielectric ones through hydrogenation reaction [42]. Recently, a scheme was proposed to modify the imaginary part of the complex refractive index of a dielectric by ion implantation [141].

Liu’s research group has a series of research on magnesium-based dynamic metasurfaces. Recently, they have demonstrated dynamic metasurface holograms based on manipulation of addressable subwavelength pixels at visible spectrum, shown in Figure 6(c). Such metasurface can be used for advanced optical encryption or information processing by controlling hydrogen, oxygen, and reaction duration, or by switching the helicity of incident light [139]. Remarkably, the refractive index of lead halide perovskites CH_3_NH_3_PbX_3_ (MAPbX_3_) and the extinction coefficients of TiO_2_ can be tuned by anion exchange and ion implantation, respectively [140, 141]. MAPbBr_3_ perovskite-based metasurface was first demonstrated to generate full phase control of 0–2*π* and high reflection efficiency. Thus, the constructed meta-hologram can be switched between “ON” state and “OFF” state by simply converting MAPbBr_3_ into MAPbI_3_ via anion exchange in a chemical vapor deposition tube [see Figure 6(d)]. In addition, Wu et al. develop a CMOS-compatible technique for ion implantation to reversibly convert TiO_2_ metasurfaces to black TiO_2_ metasurfaces. The dynamic TiO_2_ metasurface controlled by the conversion time has experimentally demonstrated an interesting potential for photochemistry application [141].

### 3.2 Stimuli to change structure reconfiguration

Directly changing the configuration of the metasurface is the straightest way to achieve tunability. For example, the planar metalens integrated with Micro-Electro-Mechanical System can easily adjust the focal point [106, 142]. The more popular reconfiguration system is to fabricate metasurfaces on stretchable or flexible substrates [143], [144], [145], [146], such as polydimethylsiloxane. Through the controllable mechanical stretching, the structural parameters of metasurfaces can be changed, thus affecting the optical resonance coupling between meta-atoms and, in-fine, achieving the manipulation of the overall optical response.

**Figure 7:**
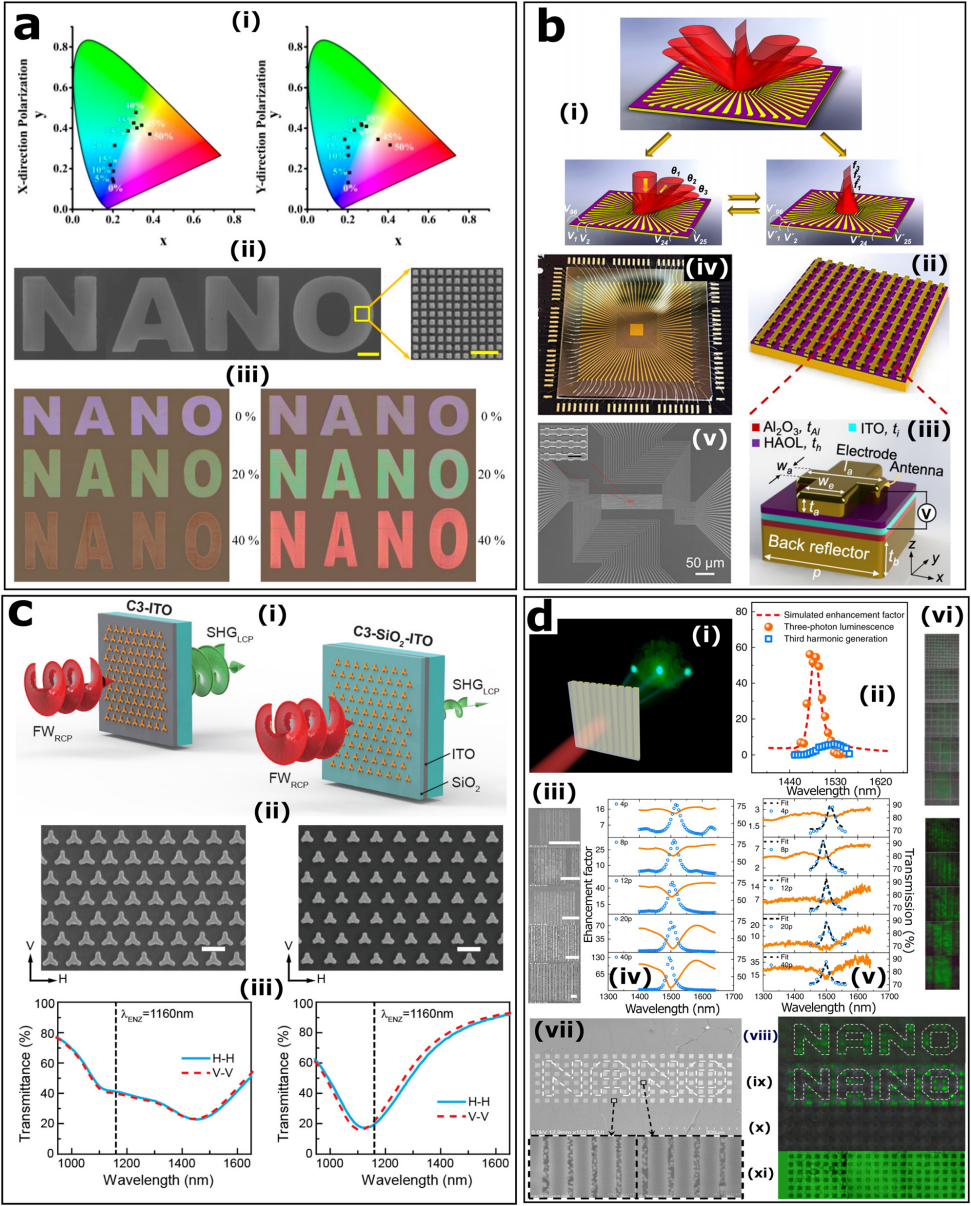
Reconfigurable metasurfaces and nonlinear metasurfaces. (a): (i) Tunable structural colors of *x*-polarized light and *y*-polarized light with varying strains in the CIE 1931 color map. (ii) SEM image of the “NANO” pattern and the partial magnification of the microstructure. Scale bar: 50 µm (left), 1 µm (right). (iii) Optical images at *x*-polarized (left) and *y*-polarized (right) light with different strains. Reproduced with permission from ref. [147]. Copyright 2019, American Chemical Society. (b): (i) Schematic of the metasurface with switchable functions, i.e., dynamic beam steering and cylindrical metalens with reconfigurable focal length. Schematic of periodic array (ii) and unit cell (iii) of the antenna elements. (iv) Photograph of the metasurface with 96 independently addressable elements. (v) SEM images of the addressable metasurface. Scale bar: 50 µm, and 500 nm for the insets. Reproduced with permission from ref. [150]. Copyright 2020, American Chemical Society. (c): (i) Schematic of SHG from two hybrid plasmonic-ENZ metasurfaces. The right one has a SiO_2_ layer. (ii) The corresponding SEM images. Scale bar: 500 nm. (iii) Transmission spectra of the metasurfaces measured for horizontally (H) and vertically (V) polarized light in the range of fundamental wave. Reproduced with permission from ref. [152]. Copyright 2020, American Chemical Society. (d): (i) Schematic of the nonlinear characterization of MAPbBr_3_ perovskite metasurfaces. (ii) The enhancement factor of three-photon luminescence and THG. Perovskite metasurfaces with different period numbers: (iii) SEM images (scale bar: 5 µm), (iv) simulated and (v) experimental results of transmission spectra and enhancement factors of three-photon luminescence, (vi) the common (up) and three-photon (down) fluorescent microscope images. The designed perovskite metasurface for nonlinear imaging: (vii) SEM images (scale bar: 300 µm, and 1 µm for the insets), (viii–xi) photoluminescence images under different pumping wavelengths of 1500, 1400, 1350, and 400 nm. Reproduced from ref. [153] under a Creative Commons Attribution 4.0 International License, Springer-Nature.

The conventional stretchable metadevices usually produce a polarization-sensitive response due to the anisotropic deformation induced by stretching, which sorely limits their practical applications. In this context, Zhang et al. carry out a polarization-insensitive stretchable TiO_2_ metasurface [147]. As shown in Figure 7(a), the authors took advantage of the different mechanisms of electric dipoles (ED) and magnetic dipoles (MD), namely the periodic effect and the near-field mutual interaction. These mechanisms will lead to resonance wavelength shifts of both ED and MD. Such metasurface can be applied to the dynamic erosion and the restoration of color information.

The category of reconfigurable metasurfaces is maturing and developing [36]. It partly includes metasurfaces that can tune optical response by mechanical deformation. This category also refers to metasurfaces that can arbitrarily control and tune the electromagnetic field at the pixel level, which acts as a perfect spatial light modulator to some extent. Digital coding metasurfaces for GHz and THz waves proposed from the perspective of information theory [36, 148] is more in line with the term reconfigurable. The coding method allows electromagnetic phase and amplitude modulations in both spatial and time domains, enriching the methodology and theory of metasurfaces [35, 149]. While introducing the concept of digital coding metasurface into the range of visible wavelength seems currently impossible, researchers did not give up and the studies of addressable metasurface are a good start. For instance, Atwater’s research group proposed a state-of-the-art prototype multifunctional metasurface that can operate at near-infrared wavelengths, as shown in Figure 7(b). Their scheme consists of 96 independently addressable elements that enable dynamic wavefront control through a pixel-by-pixel reconfiguration [150]. By altering the gate voltages applied to individual metasurface elements, the function of the reprogrammable metasurface can be switched between beam steering and light focusing. In the same year, this metadevice was optimized to improve the performance of beam steering via an inverse design strategy [151]. Such multifunctional metasurface is expected to expand into a two-dimensional phased array architecture and to be integrated on-chip electro-optical devices for light detection and ranging systems.

### 3.3 Stimuli to change the nonlinear optical response

Nanostructures are usually accompanied by strong local fields, which naturally facilitate the excitation of nonlinear effects. The diverse nonlinear effects will bring more possibilities for the realization of functional metasurfaces. Particularly, the nonlinear optical response has obvious advantages in the modulation speed, as discussed in Section 3.1.1, which provides a basis for exploring the application of all-optical high-speed switching. So far, many nonlinear processes based on metal, hybrid, and all-dielectric nanostructures have been studied [38], [39], [40], [41, 52, 154]. For example, the combination of plasmonic metasurface and multi-quantum-well heterostructures significantly enhances the nonlinear response [155], whereas the magnetic Mie resonances of Si nanodisks induce the ultrafast optical switching of 65 fs [156]. In light of this, it can be affirmed that the pursuit of large nonlinear response and ultrafast modulation speed are the major purposes of studying nonlinear effects.

Recent research has demonstrated that the second-order nonlinearity of ENZ medium can be significantly enhanced, and the corresponding second-harmonic generation (SHG) can be excited at normal incidence by integrating a plasmonic array with ITO thin-film, as illustrated in Figure 7(c) [152]. The plasmonic nanostructures in the hybrid metasurface provide strong normal components of the electric field to the ENZ substrate, which is the main reason for SHG enhancement. Additionally, the polarization of the harmonic light depends on the symmetry of plasmonic nanostructures. Interestingly, lead halide perovskite, as a non-conventional nonlinear material, can also exhibit considerable specific nonlinear effects, such as three-photon luminesce [153]. Compared with the third-order harmonic generation, the three-photon luminescence generated by the resonant MAPbBr_3_ metasurfaces can reach an enhancement factor of 60, as shown in Figure 7(d). Besides, this process is less affected by the material loss. Fan et al. designed an optical encryption from a nanostructured perovskite metasurfaces, where the encoded information is only observable once the three-photon pumped is on resonance [153]. The same research group then utilized the quasi-bounded states in the continuum (quasi-BIC) modes of perovskite metasurfaces to realize a vortex microlaser with dynamic switching of beam polarization. They proved that the modulation speed can reach the same level as that of a nonlinear all-optical switch [157, 158]. The combination of resonance modes and metasurfaces will bring rich application fields to be explored in the near future.

## 4 Tunable multifunctional metasurfaces

With the rapid development in this research field, introducing active materials into multifunctional metasurfaces is foreseen. The works that combine multifunctional and tunable metasurface together are mainly demonstrated in GHz and THz waves and implement programmable metasurface [159], [160], [161]. Related work has also been extended to visible and near-infrared wavelength range. The porotypes of addressable metasurfaces based on liquid crystals [134] and nanoelectrodes [150] have shown potential applications in the combination of tunable beam steering and zoom lenses. However, the further breakthrough has been achieved in the tunable metasurfaces that combines color nanoprinting and metahologram [109], [110], [111, 162].

**Figure 8:**
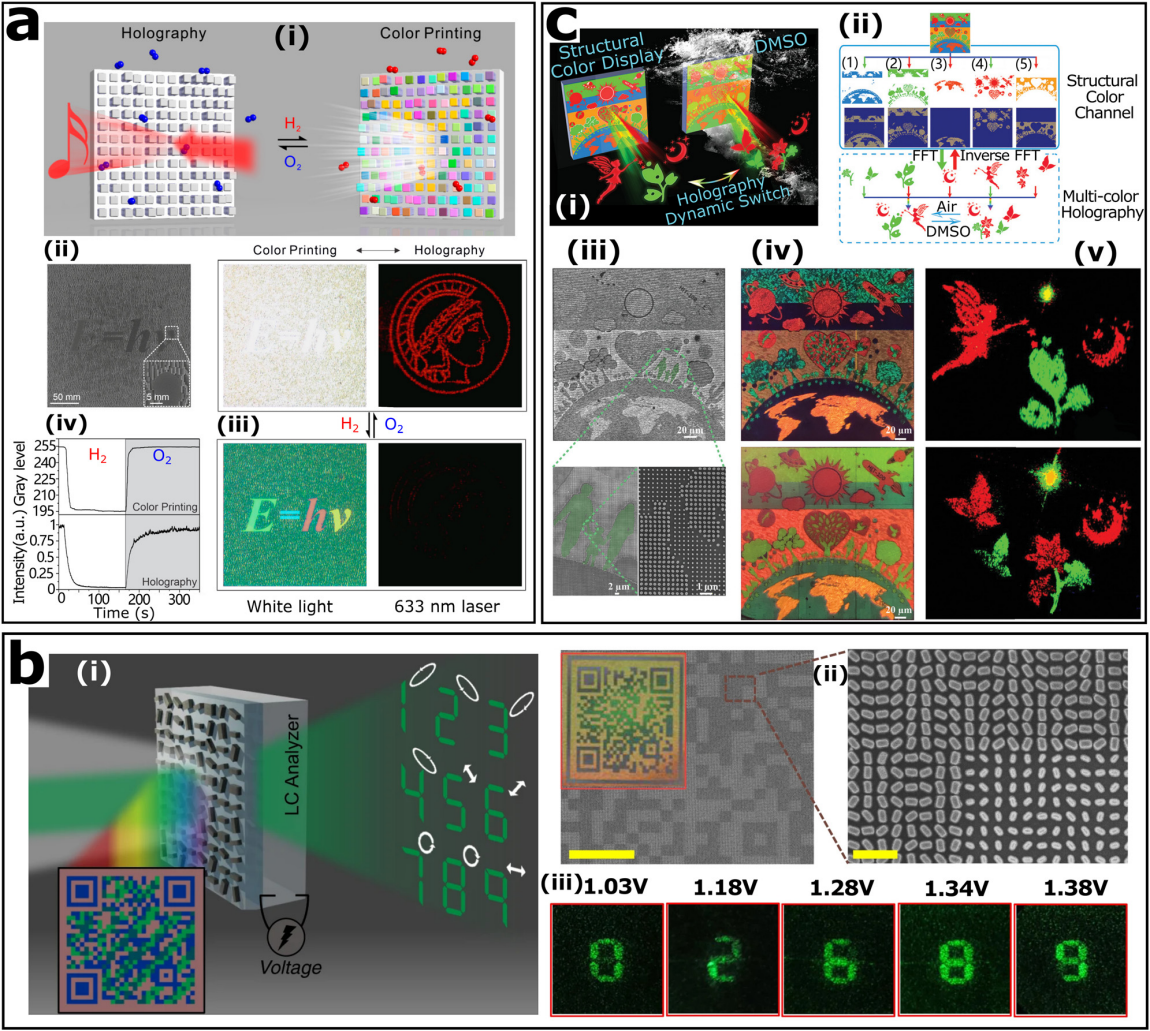
Tunable metasurfaces with a combination of color nanoprinting and metahologram. (a): (i) Schematic of the bifunction switching by hydrogenation and dehydrogenation. (ii) SEM images of the metasurface. (iii) Experimental results of switching between color print and holography. (iv) The gray value of the color print and the hologram intensity varies with time during hydrogenation and dehydrogenation. Reproduced from ref. [111] under a Creative Commons Attribution (CC-BY) License, Copyright 2020, American Chemical Society. (b): (i) Schematic of a pixelated metasurface with a two-colored QR code image and tunable vectorial holographic color prints. (ii) Optical (inset) and SEM images of the fabricated metadevice. Scale bar: 50 µm (left), 1 µm (right). (iii) Vectorial metaholograms tuning by different voltages of LCs. Reproduced from ref. [110] under a Creative Commons Attribution 4.0 International License, Springer-Nature. (c): (i) Schematic of the dynamic bifunctional metasurface. (ii) The corresponding working principle. (iii) SEM image of the metasurface. (iv) The color image (top) and holographic image (bottom) in air. (v) The corresponding images in dimethyl sulfoxide. Reproduced with permission from ref. [109]. Copyright 2021, WILEY-VCH.

Switching the two aforementioned functions through a tunable scheme was, as expected, the first design to be conceived and achieved. The spatially arranged stepwise nanocavity pixels can control both amplitude and phase of light. The obtained metasurface is able to dynamically switch between holography and color nanoprinting through the reversible phase transitions of magnesium hydrogenation and dehydrogenation, as shown in Figure 8(a) [111]. Introducing more complex design, the color nanoprinting and vectorial holography can be dynamic switched [110]. As shown in Figure 8(b), with the help of an integrated LC layer, the bifunctional metasurface achieves dynamic control of holograms, while avoiding the dependence on external polarizer and retarder. Although only the function of holography can be tuned, the vectorial holography that relies on polarization of incident light will bring more information channels, which is vital for optical encryption. Further research also simultaneously tune the color nanoprinting and the holograms by varying the surrounding medium, as shown in Figure 8(c) [109]. The destructive interference and optical resonance of dielectric nanostructures sensitively depends on the refractive index of the environmental media. It is worth noting that the latter two metasurfaces are based on silicon nanostructures, the high refractive index of silicon guarantees tunable multifunctional metasurface with high efficiency.

## 5 Conclusions and perspectives

In recent years, we have witnessed the high-speed development of metasurfaces. While smartphones have almost replaced cellular phones and short video applications have squeezed the print media market, the fate of multifunctional and tunable metasurfaces will likely overcome the static monofunctional counterparts. In this review, we firstly summarized the design schemes of multifunctional metasurfaces and the realization methods of tunable metasurfaces. We then introduced several recent studies on the metasurface operating at visible wavelengths with both multifunctionality and tunability. Here, a summary table (Table 1) is presented to systematically compare the typical research related to multifunctional and tunable metasurfaces.

**Table 1:** Comparison of typical studies to achieve multifunctional and tunable metasurfaces.

Types	Channels	Patterns	Working wavelengths	Efficiencies	Applications	Refs
Multifunctional	Wavelength	Interleaved	Visible (480, 550, 620 nm)	––	Lens	[20]
	Polarization	Interleaved	Visible (530, 640 nm)	9% (640 nm)	Holograms	[81]
					OAM beams	
	Wavelength, polarization	Non-interleaved	Visible (488, 532, 633 nm)	49% (633 nm)	Holograms	[70]
	Wavelength, polarization	Non-interleaved	Visible (450, 532, 635 nm)	52.3% (total, 45° polarization)	Holograms	[71]
	Incident angle, wavelength, polarization	Non-interleaved	Visible (470, 488, 532, 633, 785 nm)	––	Nanoprinting	[87]
	Diffraction orders	Non-interleaved	Visible (637, 685 nm)	––	Beam shaping	[23]
					External cavity laser	
	Wavelength	Multilayered	Visible (450, 550, 650 nm)	5.8–8.7% (each wavelength)	Lens	[24]
	Polarization	Multilayered	Visible (520 nm)	70% (lens 1), 60% (lens 2)	Lens	[79]

Types	Stimuli	Active materials	Working wavelengths	Modulation speed	Efficiencies	Applications	Refs
Tunable	Optical	ITO	Infrared (1180–1560 nm)	< 1 ps	––	Nonlinearity	[122]
	Thermal	GST	Mid-infrared (3.1 μm)	2 min	10% (c-GST)	Beam steering	[116]
					5% (α-GST)	Lens	
	Reconfigurable (microfluidic)	Surrounding (air, water, DMSO, CS_2_)	Visible	16 ms	––	Nanoprinting	[138]
	Chemical (hydrogenation)	Mg	Visible (633 nm)	∼1000 s	––	Holograms	[139]
						Encryption	
	Chemical (anion exchange)	MAPbX_3_	Visible (633 nm, holograms)	>10 s	22% (hologram)	Polarization conversion	[140]
					45% (polarization conversion)	Anomalous reflection	
						Holograms	
	Reconfigurable (stretchable)	PDMS	Visible	––	––	Nanoprinting	[147]
	Reconfigurable (electrical)	Addressable units	Infrared (∼1500 nm)	––	<20% (reflectance)	Beam steering	[150]
						Focusing	

Types	Channels/stimuli	Patterns/active materials	Working wavelengths	Modulation speed	Efficiencies	Applications	Refs
Tunable multifunctional	Wavelength/chemical (hydrogenation)	Non-interleaved/Mg, Pd, Ti	Visible (633 nm, holograms)	∼50 s	58% (hologram)	Nanoprinting	[111]
						Holograms	
	Polarization/electrical	Non-interleaved/LCs	Visible (532 nm, holograms)	––	––	Nanoprinting	[110]
						Holograms	
						Encryption	
	Wavelength/reconfigurable (microfluidic)	Non-interleaved/Surrounding (air, DMSO)	Visible (460, 510, 575, 633 nm, holograms)	16 ms	2.4% (red), 1.7% (green) (Holograms in DMSO)	Nanoprinting	[109]
						Holograms	

The development of static monofunctional metasurfaces is maturing, but multifunctional metasurfaces are still striving for less signal crosstalk and more integrated channels. Besides, tunable metasurfaces are seeking wider operation bandwidth and faster modulation speed. As for the research focusing on the tunable multifunctional metasurface operating at visible wavelengths, it is just getting started, so there remains a lot of space to explore.

Here, we would like to share some of the directions that are worth exploring in the future, based on our perspectives:

1. Design methods. Matrix Fourier optics enables polarization multiplexing in holograms [77]. The configuration of multilayer metasurfaces can bring more possibilities to metalens [59]. The topology-protected BIC of metasurface makes the ultrafast tuning output laser polarization possible [157]. The meta-holographic artifacts can be suppressed by fine-tuning the spatial coherent of illumination [163]. Near-field coupling engineering to control the optical response of metasurfaces [164], [165], [166]. Innovative design methods together with appropriate physical mechanisms may further expand the field of metasurface applications.
2. Material platforms. Artificial nanostructures cannot be constructed without material platforms, and conversely, the characteristics of materials will seriously restrict the performance of metadevices. Exploring new properties of emerging material platforms, such as large optical nonlinearity of ITO in its ENZ wavelengths [119] and self-cleaning ability of TiO_2_ metasurfaces by illuminating UV light [167], or finding new material platforms, such as silicon nitride [168] and gallium nitride [169], is one of the fundamental approaches to solve the inherent limitations of optical metasurface.
3. Real intelligent reconfiguration. The proposal of digital coding metasurface lays a foundation for the realization of arbitrary control of electromagnetic fields [149]. Such reconfigurable metasurface shows strong full-space and real-time control capability in GHz and THz range. In addition, combined with artificial intelligence technology, software-defined metasurface becomes more attractive [36, 37]. However, overcoming the limitations of materials and nanofabrication techniques to achieve truly reconfigurable metasurfaces in the visible range is a challenge.
4. Specific application fields. It is not realistic to replace conventional optical elements extensively with metadevices, so its application in special fields should be sought to make up for the deficiency of conventional optical elements. For example, in the emerging fields of virtual reality display [170] and photonic neuromorphic computing [128], the development of metasurfaces with multifunctionality and tunability is particularly promising.

Overall, developing metasurface with both multifunctionality and tunability is a multidisciplinary field that relies on new discoveries in basic research fields such as materials and physics, while always seeking breakthroughs in industrialization.
